# Laboratory investigations into the origin of *Mycoplasma synoviae* isolated from a lesser flamingo (*Phoeniconaias minor*)

**DOI:** 10.1186/s12917-016-0680-1

**Published:** 2016-03-12

**Authors:** Salvatore Catania, Federica Gobbo, Ana S. Ramirez, Davide Guadagnini, Elisa Baldasso, Maria Luisa Moronato, Robin A. J. Nicholas

**Affiliations:** Istituto Zooprofilattico Sperimentale delle Venezie, viale dell’Universita’ 10, Legnaro, 35020 Padova, Italy; Università degli Studi di Padova, Dipartimento di Medicina Animale, Produzioni e Salute (MAPS), Legnaro, Padova, Italy; Unidad de Epidemiología y Medicina Preventiva, Facultad de Veterinaria, Universidad de Las Palmas de Gran Canaria, C/Trasmontala s/n, Arucas, 35413 Islas Canarias Spain; Parco Faunistico LE CORNELLE, Valbrembo, Bergamo, Italy; Nutshell Lane, Farnham, Surrey GU9 0HG UK

**Keywords:** *Mycoplasma synoviae*, *Vlh*A, Lesser flamingo, *Phoeniconaias minor*, Aereosacculitis

## Abstract

**Background:**

The role of wild birds in the transmission and spread of mycoplasmas is not clear. Up to now different *Mycoplasma* species have been isolated from wild birds many of which are not considered pathogens sensu stricto for domestic flocks. This report describes the first isolation of *Mycoplasma synoviae* in a captive lesser flamingo (*Phoeniconaias minor*) held in a zoo in Italy and the laboratory investigations performed to elucidate its origin. Results showed that the strain was similar to the MS-H vaccine strain using the *vlh*A methods although no vaccination with this product was used in the zoo.

**Case presentation:**

This paper describes investigations into a case in which 10 of 12 adult lesser flamingos (*Phoeniconaias minor*) died after having recently been moved from the Netherlands to a new zoo in Northern Italy. While most of the birds appeared to have died from the stress of movement and poor adaptation to their new environment, *Mycoplasma synoviae*, an important poultry pathogen in the layer and meat industry, was isolated for the first time from the trachea of one animal presenting catarrhal tracheitis and fibrinous airsacculitis. Genetic analysis of the conserved region of the *vlhA* was not able to differentiate the flamingo strain from the MS-H vaccine strain. However differences in the sequences of the *obg* gene of the flamingo and vaccine strain were detected. A test for temperature-sensitivity (*ts*) gave a *ts*^−^ phenotype for the flamingo strain, in contrast to the *ts*^*+*^ status of the MS-H strain. Based on this information and knowing that the flamingos were not vaccinated against *M. synoviae*, it is highly likely that the flamingo was infected with a genetically similar wild strain by contact with infected birds.

**Conclusions:**

This case provides evidence for the potential role of international trade of ornamental birds as a possible route of introduction of new mycoplasma strains between countries, and moreover highlight that *vlh*A gene sequencing was not sufficient to discriminate the wild strain isolated from the flamingo from the MS-H vaccine strain.

**Electronic supplementary material:**

The online version of this article (doi:10.1186/s12917-016-0680-1) contains supplementary material, which is available to authorized users.

## Background

*Mycoplasma synoviae*, an important pathogen of poultry, can cause respiratory and articular disease, loss of production in the layer and meat industry [1]. In the poultry sector, control of mycoplasmosis is mainly based on the production and maintenance of mycoplasma-free breeders, with high levels of biosecurity measures applied to the flocks.

The role of wild birds in the transmission and spread of mycoplasmas is still unknown, despite the isolation of different *Mycoplasma* species from several avian species [2–8]. These include *M. gallinaceum, M. iners, M. pullorum, M. columbinum, M. anatis, M. sturni, M. buteonis, M. falconis, M. gypis, M. corogypsi* and *M. neophronis*; however none of these are considered pathogens sensu stricto for domestic flocks [9]. In contrast, some reports described infections of wild birds with pathogenic mycoplasmas of poultry, the most important of which is the infection of house finches (*Carpodacus mexicanus*) with *M. gallisepticum* in North America, causing a severe outbreak of conjunctivitis [10, 11]. However, the strain involved in these outbreaks was genetically different from vaccine, reference or field strains of *M. gallisepticum* isolated from commercial poultry [12].

In an epizootiological study of over 560 wild birds in Spain, *M. synoviae* was isolated from the tracheal and oropharynx samples of one red-legged partridge and three house sparrows [2]. Furthermore, *M. meleagridis* was isolated from captive falcons in the Middle East [13] and from free-ranging birds of prey in Europe [14]. In the latter case, infection could have arisen from a contaminated waste disposal site. Recently some authors have reported for the first time the isolation of *M. iowae* from the Eurasian stone-curlew (*Burhinus oedicnemus*) found in Gran Canaria [15]. More research is needed to understand the role of wild species in the maintenance and spreading of different strains of poultry pathogens species.

The lesser flamingo (*Phoeniconaias minor*) is a gregarious and social bird, very much appreciated by most ornithologists for its elegance. It is mainly distributed in Africa particularly in Tanzania, Kenya, Namibia, Botswana and South Africa, but there are also some breeding sites in south western and southern Asia. Several groups of this species are kept in captivity in the collection of some zoos worldwide [16]. Because of the particular reproductive behaviour of flamingos, breeding in captivity is difficult. In this report we describe the first isolation of *M. synoviae* from a captive lesser flamingo and investigate the variability of strains involved in the infection.

## Case presentation

At the end of April 2010 a group of 12 adult lesser flamingos was moved from the Netherlands to a new zoo in northern Italy. These birds were kept in a large zoological garden enriched with a pond. The pond water was changed completely every week and the concrete surfaces were cleaned to avoid any growth of algae. The feeding program applied was considered appropriate for this species and birds received the same diet prescribed by the previous owner with the addition of a specific dietary supplement to supply birds during the critical and stressful phases.

The aviary in which the new birds were introduced already contained other flamingos but no aggressive behaviour or dominance interactions were seen. No other birds were present in the aviary. A few days after introduction, all new birds became sick, showing wet and ruffled feathers, hypomotility, lethargy, apathy, weakness, hypothermia and poor adaptation to the new environment. After 5 days some of the new birds started to die despite the administration of antibiotic treatment with enrofloxacin (100 mg/1 kg of moist food for 14 days) and mortality continued for some days. In total 10 out of 12 flamingos died showing similar clinical signs before death. These clinical signs and mortality are frequent findings in the acclimatizing phase of wild birds to captivity when this was considered common practice.

### Materials and methods

Four carcasses of lesser flamingos were submitted to the Diagnostic Unit of Istituto Zooprofilattico Sperimentale delle Venezie, Legnaro (PD, Italy) for necropsy examination and other diagnostic procedures. Several tissues (liver, intestine, lung, air sac and brain) were collected and submitted for bacteriological test; liver, intestine and foot pad lesion were collected for virological investigations and finally air sac was submitted for histopathological and mycological examinations.

The specimens were cultured for routine microbiological procedure both aerobically and anaerobically at 37 ° C. Tracheal swabs were collected from all carcasses and submitted for mycoplasma isolation using a selective broth medium for avian mycoplasmas (Mycoplasma Experience [ME], Reigate, UK) and incubated at 37 ° C under CO_2_ conditions for at least 15 days. During this time the cultures were checked daily and when a colour change or turbidity was seen, the broth was inoculated onto ME semi-solid medium, if no change was seen after 15 days, an aliquot of the broth was inoculated onto ME semi-solid medium (ME, Reigate, UK). The inoculated agar plates were checked for mycoplasmas daily for 15 days, after this period samples were considered negative. In order to identify the *Mycoplasma* species, DNAs were extracted from broths of suspect samples, and a 16S-rDNA PCR and denaturing gradient gel electrophoresis (DGGE) method was performed [17]. The results were also confirmed with a specific PCR for *M. synoviae* [18]. Moreover in order to confirm the purity of MS we performed the 16S rDNA identification. Briefly PCR products were cleaned-up using the Performa DTR Ultra 96-well kit (Edge BioSystems, Gaithersburg, MD). Treated PCR products were sequenced using BigDye Terminator v3.1 cycle sequencing kit (Applied Biosystems, Foster City, CA) in a 16-capillary ABI PRISM 3130xl Genetic Analyzer (Applied Biosystems, Foster City, CA). Sequence data were assembled and edited with SeqScape software v2.5 (Applied Biosystems).

In addition, the analysis of the variable lipoprotein haemagglutinin-A (*vlh*A) [19] and the *sp0B*-associated GTP binding (*obg*) [20] genes were applied to the *M. synoviae* isolates in order to detect intraspecific differences.

The following oligonucleotide primers were used in the study: obg-F (5’-GTT GAT AAA GGT GGA CCA G -3’), obg-R (5’-TTA GTG CAG ATA TCT CAA TG-3’) [21] and vlhaF (5’-ATT AGC AGC TAG TGC AGT GGC C-3’) and vlhaR2 (5’-AGT AAC CGA TCC GCT TAA TGC-3’) [19]. The positive amplified PCR products, obtained from both techniques, were then sequenced at the IZS (Venezie) where PCR products were purified with ExoSAP-IT (USB Corporation, Cleveland, OH) and sequenced in both directions using the Big Dye Terminator v3.1 cycle sequencing kit (Applied Biosystems, Foster City, CA). The products of the sequencing reactions were cleaned-up using the Performa DTR Ultra 96-well kit (Edge BioSystems, Gaithers- burg, MD) and analyzed on a 16-capillary ABI PRISM 3130xl Genetic Analyzer (Applied Biosystems, Foster City, CA, USA). Sequence data were assembled and edited with SeqScape software v2.5 (Applied Biosystems). Sequences obtained were submitted to GenBank®(NIH genetic sequence database) resulting respectively in HG4^21^742.1 (*vlhA* gene) and KJ802785 (*obg* gene) accession numbers.

In order to compare the sequences with other Italian *M. synoviae* strains the MEGA 5.05 [22], Bioedit 7.0.0 [23] and BLAST search (http://www.ncbi.nlm.nih.gov/BLAST/) were used.

Once the strain of *M. synoviae* was identified, a differential growth at two different temperature (33°, and 39.5 °C) was performed in order to elucidate its temperature-sensitive phenotype (*ts*^*−*^ or *ts*^*+*^). The capacity of the strain to replicate at different temperature was established using the *colours changing unit* (CCU) method on a 96-well plate, as described by others [24, 25]. The temperature-sensitive phenotype was attributed when a difference ≥10^3^ CCU/ml occurs in the strain cultivated at the two different temperatures.

Moreover we decided to apply an additional test using three different temperatures (30°, 37° and 42 °C) in order to create larger differences between the lower and higher temperature of cultivation. The same test was performed with the MS-H vaccine strain in order to have a control sample.

Finally the *vlhA* and *obg* PCRs were applied also to the isolates grown at the three selected incubation temperatures (30°, 37° and 42 °C). All temperature-sensitive results were summarized in the Table 1.

**Table 1:** Temperature-sensitive results. In the table are reported the growth results at different temperature expressed in UCC/ml

	Temperature –sensitive test		Additional temperature test		
	33 °C	39.5 °C	30 °C	37 °C	42 °C
IZSVe/2010/5711	1.8 × 10^4^	3.36 × 10^5^	1.155 × 10^2^	1.155 × 10^6^	1.15 × 10^2^
MS-H	1.8 × 10^8^	1.27 × 10^2^	1.15 × 10^6^	2.255 × 10^6^	0

### Discussion

**Fig. 1:**
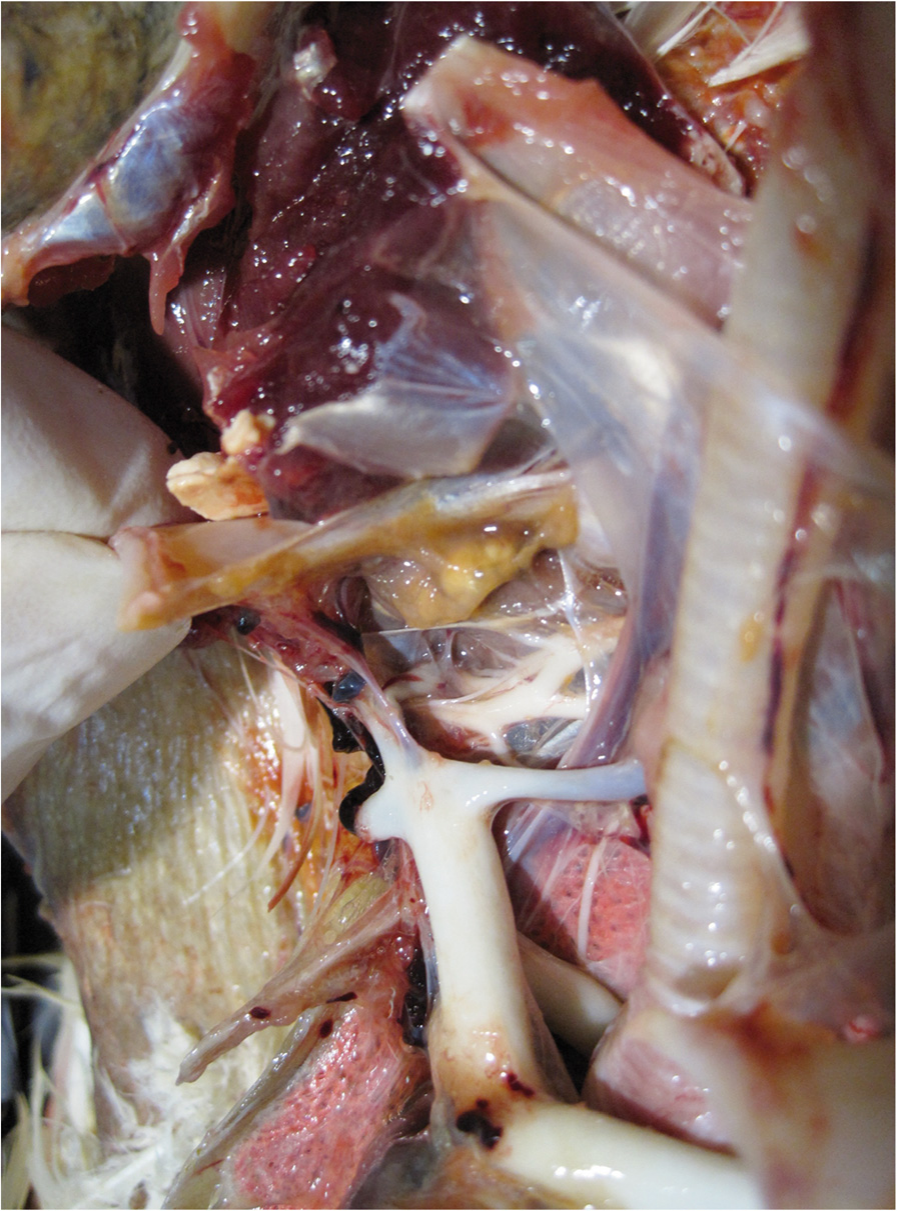
Gross pathology findings. Fibrinous airsacculitis in bird #2

Gross-pathology findings of the four birds included poor state of nutrition, absence of feed in the gastrointestinal tract and severe enteritis with some haemorrhagic intestinal areas. Only one bird (#2) showed catarrhal tracheitis and fibrinous airsacculitis (Fig. 1). All birds showed the same lesions such as chronic and septic lesions of the foot pads with joint enlargement, which were probably related to the bad conditions of the transport cage floor. Bacteriology results revealed pure cultures of *Staphylococcus spp*. (coagulase positive) from the air sac lesion as well as in the affected foot pad and *Escherichia coli* and *Clostridium perfringens* in the gut sample.

**Fig. 2:**
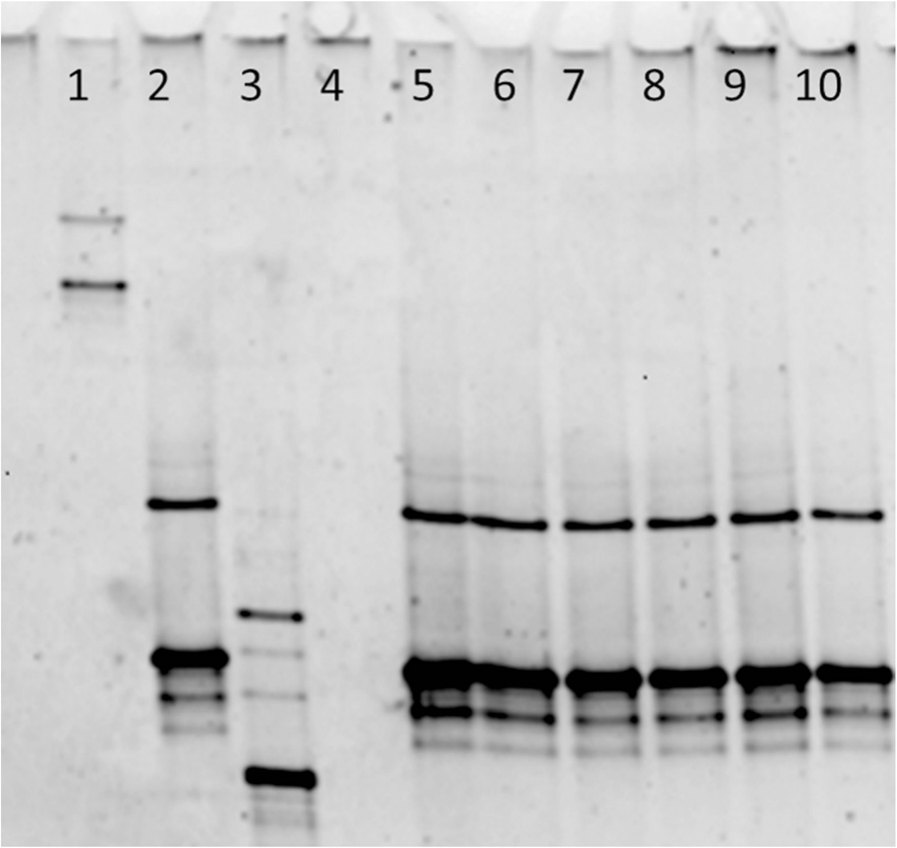
DGGE gel. In the lanes 1, 2 and 3 there are *Mycoplasma gallisepticum*, *Mycoplasma synoviae* and *Mycoplasma meleagridis* controls respectively. From lane 5 to 10 there are diagnostic samples positive for *Mycoplasma synoviae*: in lane 5 *Mycoplasma synoviae* NCTC (reference strain) and in lane 6 the strain isolated from flamingo (HG421742.1 IZSVE/2010/5711)

Mycological examination of the air sac revealed the presence of *Candida spp.* and *Rhodotorula spp.*. Moreover histopathology of the air sac showed several chronic inflammatory reactions with a necrotic centre surrounded by multinucleated giant cells (Fig. 2).

**Fig. 3:**
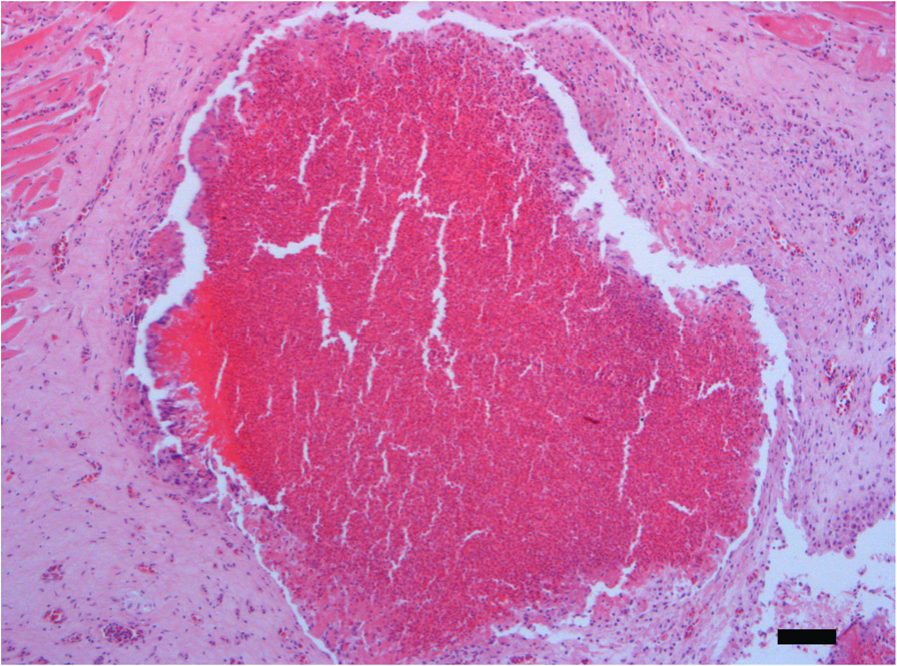
Histopathology findings. The cervical air sac is severely expanded by a focal nodular lesion composed of abundant heterophils partially surrounded by a single to double layer of macrophages and occasional multinucleated giant cells (heterophilic granuloma), the remaining tissue is diffusely enlarged by strong fibroplasia and moderate lymphoplasmacytic infiltration

After only 24 h of incubation, the ME broth culture originating from the tracheal swab of bird #2 showed a colour change and 24 h later typical mycoplasma colonies were observed on the ME agar surface. *M. synoviae* was identified by three techniques: a specific *M. synoviae* PCR [18], a specific *Mycoplasma* DGGE [17] and by sequencing of the16S gene that confirmed only the presence of *Mycoplasma synoviae*. Figure 3 shows the same DGGE profile for the flamingo isolate as other *M. synoviae* field strains, as well as the positive controls including *M. synoviae.*

After applying the PCR for *vlhA* gene to the isolate, a single amplicon was produced. This product was submitted for sequencing and the sequence obtained was compared with *M. synoviae* strains isolated in the previous year in the Italian geographical areas. The strain isolated from the flamingo did not match any Italian *M. synoviae* strains detected previously in our laboratory.

Based on the genotype classification of Bencina et al. [26] the strain was designated as genotype C subtype 3, whereas, according to the recent classification of Hammond et al., [19], the *M. synoviae* isolated from the flamingo represented a new group.

The BLAST search showed a similarity of 100 % with four *M. synoviae* strains, all isolated in Australia (GenBank® Accession n^o^: DQ661614, AY913827, AY913822, AY913823) (Additional file 1).

**Fig. 4:**
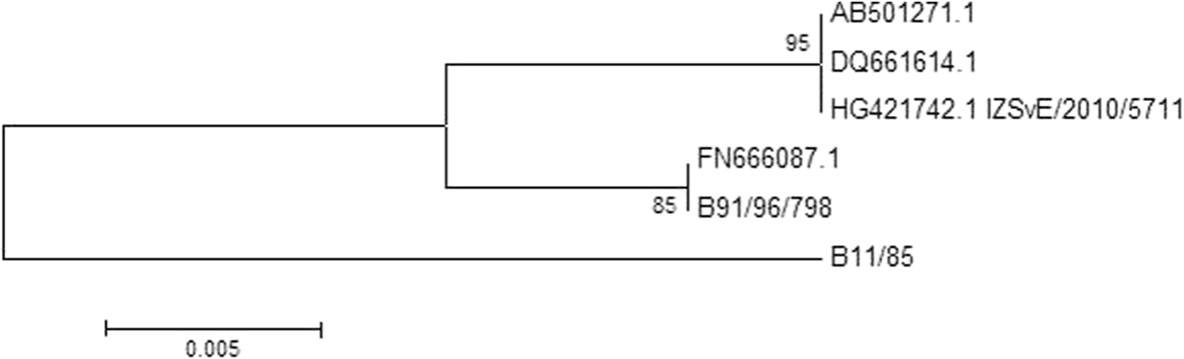
Phylogenetic analysis of the *vlh*A gene. Basing on the *vlh*A gene, the flamingo isolate (HG421742.1 IZSVE/2010/5711) clusters with the MS-H vaccine strain (AB501271) and the Australian isolate DQ661614. They are all classified as type C - subtype three by Bencina et al. [26] and group 13 by Hammond et al. [19]. The strains FN666087.1 and B91/96/798 are classified as type C3 - group 5; the strain B11/85 is a C1 – group 4. The strains FN666087.1, B91/96/798 and B11/85 have been introduced as examples of different genotypes to build the phylogenetic tree

Moreover the sequence obtained from the strain isolated from the lesser flamingo (IZSVe/2010/5711 GenBank® Accession n^o^. HG421742.1) had a 100 % of similarity in the *vlhA* gene sequence with MS-H vaccine strain (AB501271) (Fig. 4, Additional file 1).

The temperature did not affect the variability in both *vlhA* and *obg* sequences (data not shown).

The BLAST analysis of the *obg* gene showed similarity of 99 % with *M. synoviae* strains isolated in Australia (GenBank® Accession n^o^. KF875993.1, KF875997.1, KF875995, KF875994.19, [21]). Then nucleotide sequence was also compared to those investigated by Shahid et al. 2014 [21] (Additional file 2)

The *obg* gene of flamingo strain (IZSVe/2010/5711 GenBank® Accession n^o^. KJ802785) differed from others in some nucleotide positions resulting in a different sequence from the MS-H and re-isolates (*ts*^*−*^, *ts*^*+*^) and parental strain [21]. Moreover in position 367 the flamingo obg sequence presented a G.

The temperature-sensitive test demonstrated a *ts*^−^ phenotype because at 33 °C the titre was 1.8 × 10^4^ CCU/ml, whereas at 39.5 °C the titre was 3.36 × 10^5^ CCU/ml for the flamingo strain. In contrast the same test applied to the MS-H strain resulted in a titre of 1.8 × 10^8^ CCU/ml for the lower temperature, while at 39.5 °C the titre was 1.27 × 10^2^ CCU/ml. The additional test performed with a different range of temperature demonstrated a titre of 1.155 × 10^2^ CCU/ml; 1.15 × 10^6^ CCU/ml; 1.155 × 10^2^ CCU/ml at 30, 37 and 42 °C respectively. Moreover the same test applied at MS-H vaccine strain showed a titre of 1.15 × 10^6^ CCU/ml; 2.255 × 10^6^ CCU/ml at 30 °C, 37 °C respectively, whereas there was no evidence of growth at 42 °C.

## Conclusions

On the basis of gross pathology and histopathology findings it was concluded that the death of the birds was due to stress caused by movement and poor adaptation to their new captive conditions. The role of *M. synoviae* in this case, however, was not clear; indeed the air sac showed other opportunistic pathogens including fungi and bacteria such as *Staphylococcus spp*.. From the four tracheal samples, only one was positive for *M. synoviae* whereas the other three tracheal swabs were negative for *Mycoplasma spp..* It is interesting to highlight that the positive sample were isolated from the trachea of the bird with fibrinous airsacculitis. Similar findings have been reported in birds of prey where bacteria and/or fungi were isolated simultaneously with *M. meleagridis* [14]. It is also interesting to point out that this strain of *M. synoviae* grew quickly (within 24 h in vitro) unlike most strains which take many days.

The significance of the flamingo strain depends on what genetic classification system is used for *M. synoviae*. Based on Bencina’s *vlhA* scheme [26] the strain was classified as the type C3, a strain which has never been detected in Italy before as far as we are aware. However, based on Hammond’s classification [19] this would represent the first isolation of a new genotype in the EU.

Regarding the identical *vlhA* gene sequence, we examined the possibility that the flamingo strain may have derived from the temperature-sensitive (*ts*^+^) *M. synoviae* vaccine strain MS-H by differential growth at different temperatures. This showed that the flamingo strain grew poorly at low temperature which strongly suggested that it is a wild strain. However there remains the possibility that a *ts*^+^ strain could occasionally revert to a *ts*^−^ MS-H strain [20, 27, 28]. This is highly unlikely given that there was no evidence that the flamingos had been vaccinated with the MS-H vaccine. Indeed during the period of isolation the use of MS-H within Europe, in particular, Italy, was not authorised. Finally the possibility of laboratory contamination can be excluded because strict quality control tests are performed daily.

Based on our data, the diagnostic application of the *vlh*A gene analysis is of limited value for the differentiation of field and vaccine strains (Fig. 4) as was previously proposed by Ogino et al. for Japanese strains [29], and has recently suggested for European strains by Dijkman et al. (2014) [30]. Shahid et al. [25] proposed a microtitration system coupled with real time PCR to distinguish the *ts* phenotype of *M. synoviae* because in Australia the *vlhA* sequencing is often inconclusive.

Moreover, investigating the *obg* sequence variability in the flamingo, different single nucleotide polymorphisms (SNPs) were detected, allowing to better distinguishing it from related MS-H strains. Analysis showed that the flamingo strain had a G in position 367, which based on previous results [21] classified it as a field strain and not related to the MS-H strain.

The majority of *M. synoviae* strains circulating in Italy which are non-vaccine wild type field strains can be identified by the *vlhA* method (unpublished data), while the microtitration system or the analysis of *obg* gene [21] could be used to distinguish the C-type strains which in Italy account for about 10 % of the rest.

Finally this clinical case provides good evidence for possible opportunistic transmission and spread of avian pathogens, which is compounded by the international trade in ornamental or zoo birds and may represent possible vectors of avian pathogens. These types of bird live in the open-air where strict biosecurity measures are not applied, thus enabling the direct and indirect contact with autochthonous fauna, increasing the risk of spreading infections if exotic microorganisms have contact with free populations. Hence these ornamental species could play an important epidemiological role in spreading infectious disease to the industrial poultry.

## Additional files

Additional file 1: Alignment of partial *vlh*A sequences. *Mycoplasma synoviae* strains’ *vlh*A sequences showed a similarity with the lesser flamingo isolates (HG421742.1 IZSVE/2010/5711). In this figure we insert only one of the strains that showed a 100 % of similarity for the Australian isolate; its accession number is DQ661614 because the other one reported in the results showed the same sequence. The AB501271 is the *vlhA* sequence of MS-H vaccine strain. Finally, the accession number FN666087 is one of the two EU strains that showed the higher similarity. (TIF 1431 kb) Additional file 2: Alignment of partial *obg* sequences. The *obg* gene sequences of *Mycoplasma synoviae* isolated from flamingo (KJ802785 IZSVE/2010/5711) was aligned with MS-H (KC990837.1), re-isolate *ts* ^*−*^ (KC990838.1) and *ts +* (KC990839.1) and parental strain *ts* ^*−*^ (KC990840.1). Nucleotide differences are specified by the nucleotide, while dot represented no nucleotide changing. (TIF 608 kb)

## Abbreviations

DGGE: Denaturing Gradient Gel Electrophoresis
DNA: Deoxyribonucleic acid
EU: European Union
ME: Mycoplasma experience
MS: Mycoplasma synoviae
obg: spOB-associated GTP binding protein
PCR: polymerase chain reaction
SNPs: single nucleotide polimorphisms
ts: temperature sensitive
vlhA: variable lipoprotein hemagglutinin A

## References

[1] Bradbury JM, Miles R, Nicholas R (1998). Recovery of Mycoplasmas from Birds. Mycoplasma Protocols.

[2] Poveda JB, Carranza J, Miranda A, Garrido A, Hermoso M, Fernandez A, Domenech J (1990). An epizootiological study of avian mycoplasmas in southern Spain. Avian Pathology.

[3] Poveda JB, Giebel J, Flossdorf J, Meier J, Kirchhoff H (1994). Mycoplasma buteonis sp. nov., Mycoplasma falconis sp. nov., and Mycoplasma gypis sp. nov., Three Species from Birds of Prey. Int. J. Syst. Bacteriol..

[4] Pennycott TW, Dare CM, Yavari CA, Bradbury JM (2005). Mycoplasma sturni and Mycoplasma gallisepticum in wild birds in Scotland. Vet Rec.

[5] Loria GR, Ferrantelli E, Giardina G, Li Vecchi L, Sparacino L, Oliveri F, McAuliffe L, Nicholas RA (2008). Isolation and characterization of unusual Mycoplasma spp. from captive Eurasian Griffon (Gyps fulvus) in Sicily. J Wildl Dis.

[6] Lierz M, Hagen N, Hernadez-Divers SJ, Hafez HM (2008). Occurrence of mycoplasmas in free-ranging birds of prey in Germany. J Wildl Dis.

[7] Stipkovits L, Szathmary S (2012). Mycoplasma infection of ducks and geese. Poult Sci.

[8] Suárez-Pérez A, Ramírez AS, Rosales RS, Calabuig P, Poveda C, Rosselló-Móra R, Nicholas RA, Poveda JB (2012). Mycoplasma neophronis sp. nov., isolated from upper respiratory tract of Canarian Egyptian Vulture (Neophron percnopterus majorensis). Int J Syst Evol Microbiol.

[9] Brown R.B. Phylum XVI. Tenericutes Murray 1984a, 356^vp^ (Effective publication: Murray 1984b, 33). In: Bergey’s Manual of Systematic Bacteriology. 2nd Ed. Volume 4. New York Dordrecht Heidelberg London: Springer. p. 567–613.

[10] Ley DH, Berkhoff JE, McLaren JM (1996). Mycoplasma gallisepticum isolated from house finches (Carpodacus mexicanus) with conjunctivitis. Avian Dis.

[11] Fischer JR, Stallknecht DE, Luttrell P, Dhondt AA, Converse KA (1997). Mycoplasmal conjunctivitis in wild songbirds: the spread of a new contagious disease in a mobile host population. Emerg. Infect. Dis..

[12] Ley DH, Berkhoff JE, Levisohn S (1997). Molecular epidemiologic investigations of Mycoplasma gallisepticum conjunctivitis in songbirds by random amplified polymorphic DNA analyses. Emerg. Infect. Dis..

[13] Lierz M, Schmidt R, Runge M (2002). Mycoplasma species isolated from falcons in the Middle East. Vet Rec.

[14] Lierz M, Schmidt R, Brunnberg L, Runge M (2000). Isolation of Mycoplasma meleagridis from free-ranging birds of prey in Germany. J Vet Med B Infect Dis Vet Public Health.

[15] Suarez-Perez A, Ramirez AS, Rosales RS, Calabuig P, Poveda CG, Vega-Orellana OM, Mederos LE, Nicholas RAJ, Poveda JB (2010). Isolation of Mycoplasma spp., from Eurasian Stone Curlew (Burhinus oedicnemus insularum) in Canary Island. Italy, Chianciano. Proceedings of the 18th International Congress of the International Organization for Mycoplasmology IOM.

[16] ISIS. International Species Information System. 2009. http://www2.isis.org/Pages/Home.aspx

[17] McAuliffe L, Ellis RJ, Lawes JR, Ayling RD, Nicholas RA (2005). 16S rDNA PCR and denaturing gradient gel electrophoresis; a single generic test for detecting and differentiating Mycoplasma species. J Med Microbiol.

[18] Lauerman LH, Hoerr FJ, Sharpton AR, Shah SM, Van Santen VL (1993). Development and application of a polymerase chain reaction assay for Mycoplasma synoviae. Avian Dis.

[19] Hammond PP, Ramírez AS, Morrow CJ, Bradbury JM (2009). Development and evaluation of an improved diagnostic PCR for Mycoplasma synoviae using primers located in the haemagglutinin encoding gene vlhA and its value for strain typing. Vet Microbiol.

[20] Shahid MA, Markham PF, Markham JF, Marenda MS, Noormohammadi AH (2013). Mutations in GTP binding protein Obg of Mycoplasma synoviae vaccine strain MS-H: implications in temperature-sensitivity phenotype. PLoS One.

[21] Shahid MA, Markham PF, Marenda MS, Agnew-Crumpton R, Noormohammadi AH (2014). High-Resolution Melting-Curve Analysis of obg Gene to Differentiate the Temperature-Sensitive Mycoplasma synoviae Vaccine Strain MS-H from Non-Temperature-Sensitive Strains. PLoS One.

[22] Tamura K, Peterson D, Peterson N, Stecher G, Nei M, Kumar S (2011). MEGA5: molecular evolutionary genetics analysis using maximum likelihood, evolutionary distance, and maximum parsimony methods. Mol Biol Evol.

[23] Hall TA (1999). BioEdit: a user-friendly biological sequence alignment editor and analysis program for Windows 95/98/NT. Nucleic Acids Symp Ser.

[24] Morrow CJ, Markham JF, Whithear KG (1998). Production of temperature-sensitive clones of Mycoplasma synoviae for evaluation as live vaccines. Avian Dis.

[25] Shahid MA, Ghorashi SA, Agnew-Crumpton R, Markham PF, Marenda MS, Noormohammadi AH (2013). Combination of differential growth at two different temperatures with a quantitative real-time polymerase chain reaction to determine temperature-sensitive phenotype of Mycoplasma synoviae. Avian Pathology.

[26] Bencina D, Drobnic-Valic M, Horvat S, Narat M, Kleven SH, Dovc P (2001). Molecular basis of the length variation in the N-terminal part of Mycoplasma synoviae hemagglutinin. FEMS Microbiol Lett.

[27] Markham JF, Scott PC, Whithear KG (1998). Field evaluation of the safety and efficacy of a temperature-sensitive Mycoplasma synoviae live vaccine. Avian Dis.

[28] Noormohammadi AH, Jones JF, Harrigan KE, Whithear KG (2003). Evaluation of the non-temperature-sensitive field clonal isolates of the Mycoplasma synoviae vaccine strain MS-H. Avian Dis.

[29] Ogino S, Munakata Y, Ohashi S, Fukui M, Sakamoto H, Sekiya Y, Noormohammadi AH, Morrow CJ (2011). Genotyping of Japanese field isolates of Mycoplasma synoviae and rapid molecular differentiation from the MS-H vaccine strain. Avian Dis.

[30] Dijkman R, Feberwee A, Landman WJM (2014). Variable lipoprotein haemagglutinin A (vlhA) gene sequence typing of mainly Dutch Mycoplasmma synoviae isolates: comparison with vlhA sequences from Genbank and with amplified fragment length polymorphism analysis. Avian Pathol..

