# Erratum to: Laboratory investigations into the origin of *Mycoplasma synoviae* isolated from a lesser flamingo (*Phoeniconaias minor*)

**DOI:** 10.1186/s12917-016-0699-3

**Published:** 2016-04-12

**Authors:** Salvatore Catania, Federica Gobbo, Ana S. Ramirez, Davide Guadagnini, Elisa Baldasso, Maria Luisa Moronato, Robin A. J. Nicholas

**Affiliations:** Istituto Zooprofilattico Sperimentale delle Venezie, viale dell’Universita’ 10, Legnaro, Padova 35020 Italy; Dipartimento di Medicina Animale, Produzioni e Salute (MAPS), Università degli Studi di Padova, Legnaro, Padova Italy; Unidad de Epidemiología y Medicina Preventiva, Facultad de Veterinaria, Universidad de Las Palmas de Gran Canaria, C/Trasmontala s/n, Arucas, Islas Canarias 35413 Spain; Parco Faunistico LE CORNELLE, Valbrembo, Bergamo Italy; Nutshell Lane, Farnham, Surrey GU9 0HG UK

Unfortunately, during production of this article [1], an error was introduced. The labels for Figures 2 and 3 were switched, leading to an incorrect reference to each image in the text. Figure 2 should have been labelled as Figure 3 and Figure 3 should have been labelled as Figure 2. The original article has also been updated to reflect this.
