# Supplementary figures and images for: Laboratory investigations into the origin of Mycoplasma synoviae isolated from a lesser flamingo (Phoeniconaias minor)
Source: BMC Vet Res. 2016 Mar 12;12:52. doi: 10.1186/s12917-016-0680-1 (PMC4788927; doi:10.1186/s12917-016-0680-1)

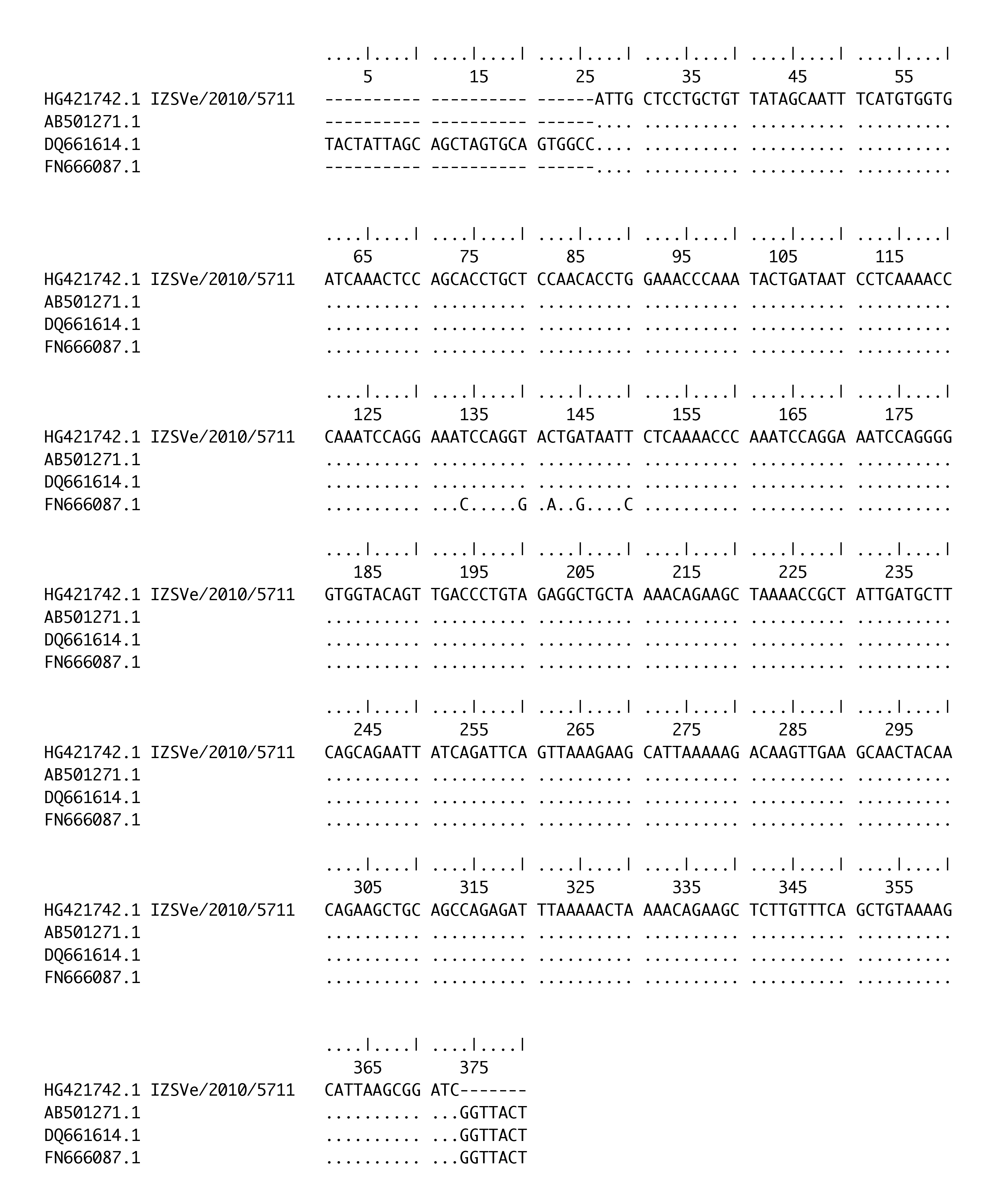

Supplement: Additional file 1: — Alignment of partial vlhA sequences. Mycoplasma synoviae strains’ vlhA sequences showed a similarity with the lesser flamingo isolates (HG421742.1 IZSVE/2010/5711). In this figure we insert only one of the strains that showed a 100 % of similarity for the Australian isolate; its accession number is DQ661614 because the other one reported in the results showed the same sequence. The AB501271 is the vlhA sequence of MS-H vaccine strain. Finally, the accession number FN666087 is one of the two EU strains that showed the higher similarity. (TIF 1431 kb) [file 12917_2016_680_MOESM1_ESM.tif]

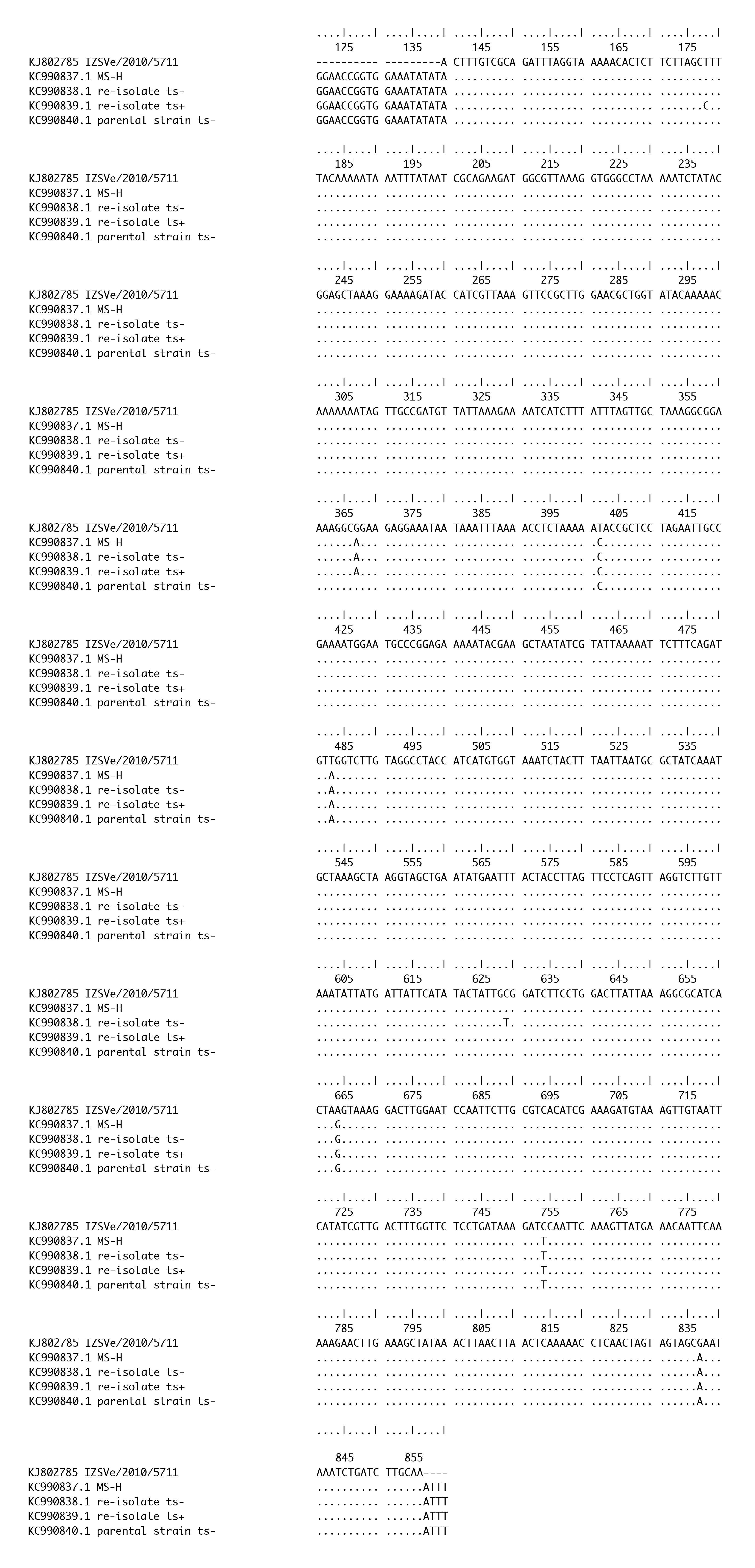

Supplement: Additional file 2: — Alignment of partial obg sequences. The obg gene sequences of Mycoplasma synoviae isolated from flamingo (KJ802785 IZSVE/2010/5711) was aligned with MS-H (KC990837.1), re-isolate ts − (KC990838.1) and ts + (KC990839.1) and parental strain ts − (KC990840.1). Nucleotide differences are specified by the nucleotide, while dot represented no nucleotide changing. (TIF 608 kb) [file 12917_2016_680_MOESM2_ESM.tif]
